# Investigation of personal data protection mechanism based on blockchain technology

**DOI:** 10.1038/s41598-023-48661-w

**Published:** 2023-12-08

**Authors:** Rongrong Zhu, Maofeng Wang, Xiaofang Zhang, Xinyun Peng

**Affiliations:** 1 School of Intelligent Engineering, Shaoguan University, Shaoguan, 512000 Guangdong China; 2 School of Information and Media, Yantai Engineering & Technology College, Yantai, 264000 Shandong China

**Keywords:** Computational science, Computer science, Information technology, Software

## Abstract

Blockchain technology is increasingly being used in personal data protection. Inspired by the importance of data security, this paper proposes a personal data protection mechanism based on blockchain, combined with distributed hash tables and cryptography, to enhance users' control over the data generated using web applications. This paper designs this mechanism's system model and describes the three aspects in detail: data storage mechanism, data encryption mechanism, and data trading mechanism. Among them, the data storage mechanism restricts user data to be stored only in the local storage space of the user terminal, the decentralized blockchain network, and the distributed hash table network to ensure that enterprises providing network applications cannot privately store user interaction data, the encryption mechanism is responsible for encrypting all user data recorded in the network and allows users to control the key of the data to ensure the security of the user data in the blockchain and distributed hash tables, the data transaction mechanism allows users to trade their data, and to incentivize enterprises to assist users in collecting personal data, data transaction contracts are built into the data transaction mechanism, allowing enterprises to receive a share of the revenue from user data transactions. Then, for data transactions, use the Stackelberg game to simulate the revenue sharing between users and service providers in data trading to incentivize enterprises providing web services to assist users in collecting their data. The simulation results show that when the number of users is 1000, the revenues of this scheme for service providers are 31%, 561%, and 19% higher than the existing scheme. Finally, the personal data protection platform is implemented by code to verify the feasibility of the theory proposed in this paper in personal data protection.

## Introduction

With the development of information technology, web services have profoundly changed the way of life of Internet users. While rich web applications have brought great convenience to users, the companies behind them also collect a lot of personal data from users. Compared to enterprises, users who are the data source lack effective control over their data, users are often forced into a "privacy-for-convenience" dilemma, where they must provide large amounts of data to enterprises in exchange for services. It is difficult for users to know what data are collected using Web services and whether enterprises use them appropriately. If enterprises leak and misuse user data, the users bear the corresponding losses.

In March 2020, Weibo said that attackers gained access to part of its database, affecting 538 million Weibo users and their personal information, including real names, website usernames, genders, locations, and phone numbers. The attackers then sold the data on the dark web for $250.00. In April 2021, a user posted a huge amount of data in a hacker forum about 533 million Facebook users in 106 countries, including information such as IDs, users' full names, locations, birthdays, bios, and email addresses. In June 2022, the phone numbers and email addresses of 5.4 million accounts on Twitter's website were compromised, allowing hackers to retrieve public information about the accounts to create user records containing both private and public information. As we can see, while Web applications bring convenience to users, they also bring a lot of security and privacy issues.

Under the current mainstream client–server or browser-server architecture, Web applications are mainly composed of a Web front-end running in the user's device and a Web back-end deployed in the enterprise server. This centralized architecture allows enterprises providing Web services to privately collect and store user-generated data without the user's authorization, and it is difficult for regulatory agencies to regulate these enterprises and the content of the data stored in the enterprise server in real-time. In response to the problem of easy backup of user data by enterprise servers, existing research at home and abroad mainly utilizes the information tracing method to restrain enterprises, i.e., when an enterprise backs up and leaks user data, it is easy for supervisory organizations to locate the enterprise that leaks user data. However, this does not change the nature of enterprises that can easily back up user data, and there may still exist some enterprises that are desperate to back up and misuse users' personal data in order to seek profits. Moreover, since users' personal data may be scattered in the servers of multiple enterprises in the course of using multiple Web services, it is also difficult for supervisory organizations to locate the specific enterprises that leaked users' data in the event of a user data leakage incident.

In recent years, blockchain technology has entered a rapid development period and has been widely applied in various fields, bringing new solutions to various application scenarios. Xu et al. proposed a multi-candidate voting model based on blockchain technology to address the issues of poor anonymity and low efficiency in traditional voting^1^. This model introduces asymmetric encryption and anonymous protection voting algorithms, which can calculate votes without relying on third parties and display voting results in real time to meet different levels of voting security requirements. In order to solve the contradiction between traceability and privacy of evidence due to a large amount of digital evidence, Tian et al. proposed a secure digital evidence framework (Block-DEF) using blockchain^2^, which adopts a loosely coupled structure where evidence and evidence information are maintained separately. Only the evidence information is stored in the blockchain, and the evidence is stored on a trusted storage platform. To avoid blockchain bloat, a lightweight blockchain is proposed that combines a hybrid block structure with an optimized name-based pragmatic Byzantine fault-tolerant consensus mechanism. In order to support the traceability and privacy of evidence, multiple signature techniques are used for evidence submission and retrieval. The framework guarantees the integrity and validity of evidence and well-balances privacy and traceability. Xu et al. designed a two-stage analysis scheme to identify and summarize the application of blockchain in modern logistics information sharing and its challenges, in response to the traditional logistics industry^3^, where information asymmetry, cargo authenticity issues, and lack of security all impede business transactions, leading to high operational costs and time-consuming problems. A Latent Dilettante Allocation Theme Model was used and 2465 studies were selected to summarize the application scenarios. These data were used to create a blockchain integration framework to identify the best applications of blockchain in modern logistics information sharing.

Compared with the centralized architecture that is difficult to supervise, blockchain technology, which is decentralized, transparent, tamper-evident, and easy to audit, is a new solution for the security protection of personal data. Blockchain technology has characteristics that can effectively meet the data security needs required for private data^4–6^. It can be used as an effective solution for personal data security problems, assisting users in monitoring the behavior of enterprises collecting user data and enhancing users' control over their data. Therefore, based on blockchain technology, designing a personal data protection mechanism to enhance users' data control ability and protect users' data generated in web services can provide a reference for further research on personal data security protection issues.

There are six sections in total. The first section is the “Introduction”, which presents the importance of personal data protection and the security issues posed by the collection of users' personal data by enterprises, and defines the research idea. The second section is the “Related work”, which analyzes the current status of research related to designing a general Web service framework to enhance the data control ability of users in the process of using Web services through blockchain technology, analyzes the shortcomings, gives its own research method, and clarifies the research framework. The third section is the “System Model”, which designs a personal data protection platform model, proposes a personal data protection mechanism and related protocols, and analyzes the system operation process and how to ensure that the data is in one's possession. The fourth section is the “Results and discussion”, firstly describes the operation process of the email application in the system and analyzes how it ensures data security. Secondly, the Stackelberg game is used to optimize the returns of the data trading process in the simulation and analysis. The fifth section is the “Design of the platform”, which designs the personal data protection platform from the perspective of software development, discussing the platform's requirement design, functional design, operation flow, and the overall framework of the platform's core. The sixth section is the “Conclusion and Outlook”, which summarizes the current work accomplished, the shortcomings, and looks ahead to future research efforts.

## Related work

Some scholars have devoted themselves to designing a more generic web service framework using blockchain technology. For example, Zyskind et al. proposed a decentralized personal data management system to solve the problem of all third-party services accessing users' data at will^7,8^. The system uses a traditional bitcoin system to send commands, such as storage commands, query requests, and shared data commands. This system allows users to control their data and authorize or revoke access to user data from all third-party services, thus eliminating users' concerns about personal privacy data issues when dealing with third-party services. Wang et al. build the data privacy protection in the system starting with the software architecture, suggest a realization method of data security sharing and business collaboration that algorithm runs instead of the direct interaction of source data, and establish data service usage guidelines that "do not require all, but use, call, and go"^9^. With a focus on the viability and simple, effective implementation, an entire architecture and method of implementation for data-efficient transactions were developed, including data three-weight separation, data privacy isolation on the chain, cross-cloud and cross-network deployment algorithm decomposition multi-center collaboration, as well as collaborative services on the chain and off the chain. Jiang et al. proposed a traceable method for personal information registration based on blockchain, which can distinguish the service provider obtained the information legally or illegally, by storing the personal information transaction records on the blockchain^10^. The proposed method includes both direct and third-party personal information transaction scenarios. In different scenarios, the user will send the encrypted personal information data or authorization file to the service provider. After the transaction record is confirmed to be on the blockchain, the certification center will assist the service provider to decrypt the personal information. With this method, users can clearly understand which personal information has been delivered to which service providers. Moreover, the user’s personal information data involving privacy are not stored on or transmitted through the blockchain. Hence, there is no additional risk of information disclosure, so as to achieve the purpose of personal information protection. Lazarovich et al. designed a secure platform for storing users' data and described how users on this platform authorize third-party web services to access users' data^11^. In this platform, the user and the web service share a key to access the user's encrypted data, and the record of access is written to the blockchain for easy monitoring by the user, thus protecting the user's privacy. Le et al. designed a web application that applies blockchain to distributed hash tables to improve the security and privacy of personal data on a distributed network. The system ensures authorized users can only see data with the correct key to decrypt the data through data encryption. Access control through smart contracts provides another layer of security checks to access the data by verifying the user's identity before granting access^12^. Combining these two approaches can improve security and reduce the risk of data theft by malicious users. Chiu et al. designed a scheme that uses smart contracts and blockchain to provide a secure data-sharing and access environment. It can address the behaviour of web service providers who can access user data at will^13^. The scheme proposes three main participants in the data access process: data demanders, data agents, and individual users, each with its key pair for encrypting and signing data. The data agent acts as the authority controller, establishing the data access authority and access process between the individual user and the data demander through a smart contract, which provides security for the data interaction between the individual and the data demander. Smita et al. proposed a new blockchain-based secure decentralized system, using distributed hash tables for secure data transfer to address the problem of dishonesty in accessing data by third-party systems^14^. In the proposed approach, the data owner uploads the encrypted file to the distributed hash table and divides it into n secret parts, called hash codes, for data security. The data owner must additionally write access rights to access this secure data. For security purposes, the system uses two levels of key management. First, the data owner encrypts the file, and then the distributed hash table generates hash codes for the encrypted file. Based on the security analysis, the proposed system is effective against individual and collaborative malicious persons and untrusted cloud servers. Alrebdi et al. propose a blockchain-based decentralized data security management platform for the insecurity of personal data access in electronic healthcare management systems^15^, which consists of a smart contract through which users can transact with the system. In addition, the system uses the Interplanetary file system (IPFS) and cloud computing to store patients' data and files. experimental results and system security analysis show that the system performs search and verification tasks securely and quickly through the network.

Most of the above research, whether they combine specific scenarios or design a general data protection framework, secure users' data by improving users' data control capabilities and constraining the control authority of web services. However, most interaction data is generated by users using web services, which are close to web businesses and need to rely on web applications for collection. There is already research that is difficult to constrain all backup data behaviours of a specific web application. The enterprise defines the logical code for collecting data, and it is easy to back up user data privately on its server. Some enterprises may risk backing up and misusing users' data to seek benefits^16–19^. It is difficult for regulators to locate the enterprise that leaked users' data. Therefore, this paper proposes a personal data protection mechanism to solve the above problems. It uses blockchain, distributed hash tables, and cryptography to secure user data using web services. Combined with the decentralized nature of blockchain, it deploys the web back-end functions in the blockchain's smart contracts and stores the data with a large amount of data in distributed hash tables^20^, jointly maintained by all nodes in the network. This joint maintenance of the web back-end network by all nodes replaces the single enterprise controlling the web back-end. It reduces the enterprise's ability to control web applications, thus strengthening the user's ability to control personal data.

## System model

### Problems and solution ideas

**Figure 1:**
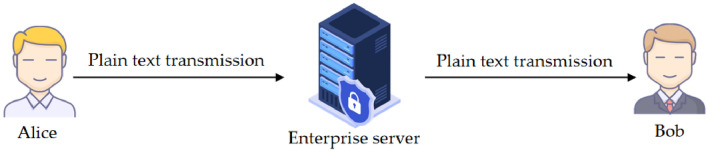
Information transfer in the client–server architecture.

Figure 1 shows the typical C/S architecture. The interaction data generated by users in web applications will inevitably flow through the web back-end for processing. Since the web back-end runs in the enterprise's internal server, it can collect and store user data without authorization. The secrecy of enterprise servers makes it difficult for regulators to monitor the data content. Users cannot know what personal data the enterprise has collected and used.

**Figure 2:**
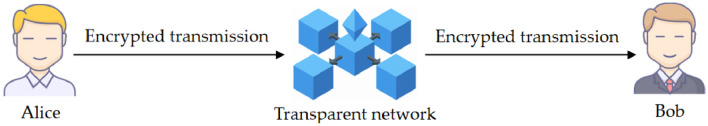
The data transmission in a transparent network.

Users need to have control over their data, easy access to their data, and decide what private data not to share with enterprises. Therefore, it is necessary to make it mandatory that the web back-end runs openly and transparently, not be controlled by a single enterprise. Under this solution, the web back-end program provided by the enterprise is public to the external network. The external network can easily supervise whether the enterprise generates codes that endanger the user's security, thus safeguarding the user's data. As shown in Fig. 2, if the web runs in a transparent network maintained by multiple nodes, all other nodes can supervise the behaviour of the web back-end, and the web back-end will not be able to generate behaviours that are detrimental to users.

### Platform architecture

#### Blockchain

Blockchain technology is a decentralized distributed database, and the data is stored to be verified by all blockchain nodes without trust relationships^21^. Decentralization means that there is no control organization like a central institution in the whole network, and every node has the same status and authority. The problem of a node will not lead to paralysis. De-trusting means no need for a third-party centralized organization in the system and no need to establish any trust relationship between nodes. The blockchain system is open and transparent, trading is traceable, and no data can be tampered with^22–24^.

#### Smart contracts

Smart contracts are just like the contracts we sign in reality, with the characteristic that whenever a contract is created, its contract code is fixed and cannot be changed. In a blockchain, a smart contract is a computer program code stored and run by all consensus nodes on the blockchain, which is guaranteed to be unchangeable by the tamper-evident nature of the blockchain^25^.

#### Distributed hash table

Distributed Hash Table (DHT) is a distributed storage system class. It can store data decentralized to nodes in a distributed system^26^. In a DHT, data is stored via key-value pairs, and each peer node needs to keep only a portion of the key-value pairs. Any peer node can query this DHT by a key. The DHT will locate the peer node with that key-value pair and return that key-value pair to the querying node.

#### Personal data protection platform

**Figure 3:**
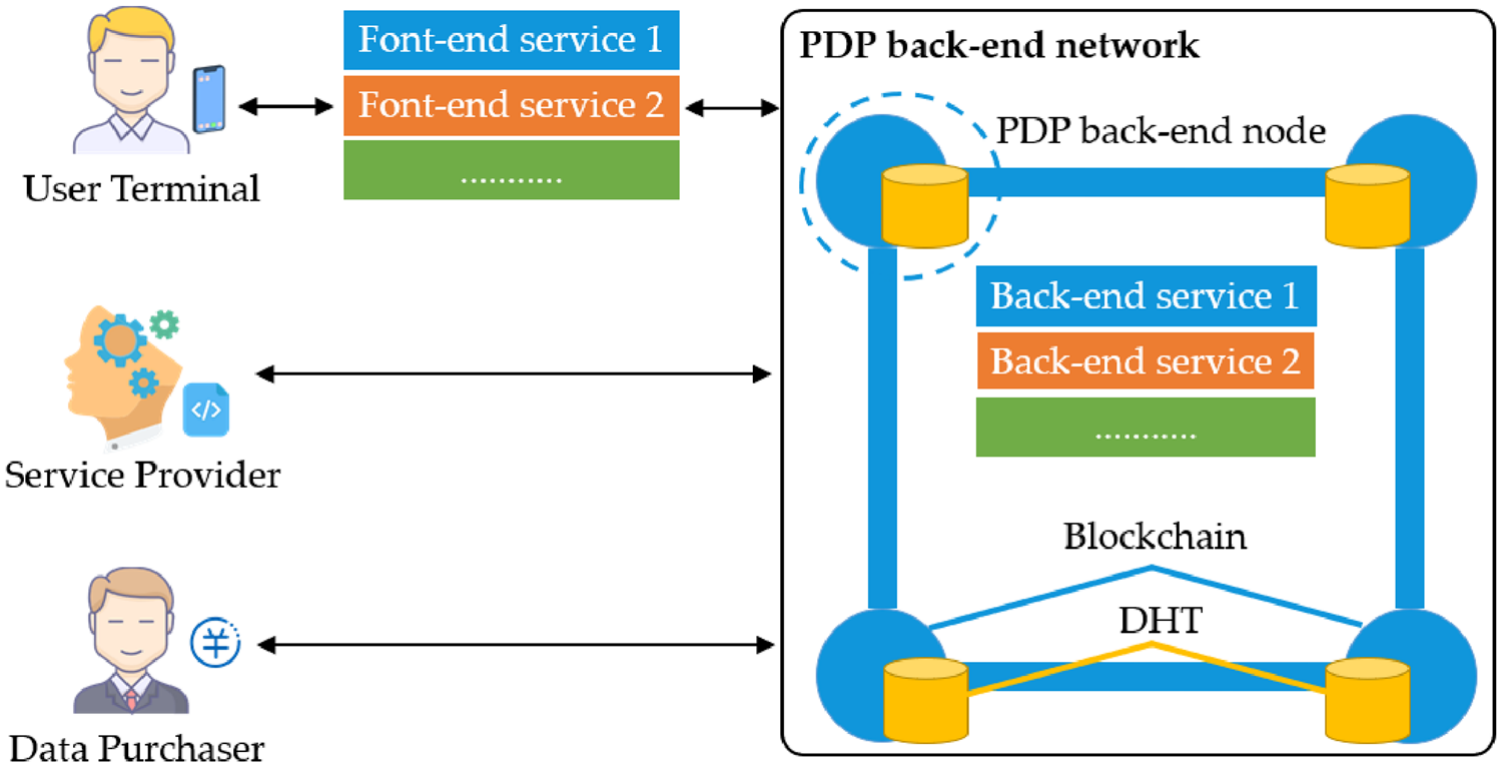
System model in the PDP platform.

The system model considered in this paper is shown in Fig. 3, consisting of service providers, web applications, user terminals, data purchasers, and a Personal Data Protection (PDP) Platform.

- *User terminal*: PDP platform and web front-end are installed in the user terminal. Users enjoy the services by using the web front-end and simultaneously generate personal data, which are collected by the web front-end. Users can trade their data on the trading page provided by the web front-end to obtain revenue.
- Service provider: Third-party developers who provide web applications for users and get revenue from trading data by users.
- *Data purchaser*: Organizations that purchase user data to complete multiple requirements, such as scientific research institutions that use personal data for scientific research.
- *Web application*: Consists of two parts, web front-end, and back-end. The web front-end is the application that interacts with users. It is located on the user terminal, providing an interactive interface and computing and storage functions. The web back-end runs in the PDP back-end network, and the program code is deployed in the blockchain network in the form of smart contracts, while other data resources are stored in the DHT network.
- *PDP platform*: An environment for web applications, providing a series of protocols. web applications conforming to the protocols can run in the PDP platform. It consists of the front-end and back-end. The Front-end runs on the user terminal and is responsible for managing the web front-end of all web applications. The back-end is a new computing and storage network combining the blockchain and the distributed hash table. It uses blockchain computing logic to achieve computing power and distributed hash tables to achieve storage power. Unlike back-end servers maintained by a single service provider, the back-end network combines the decentralized feature of blockchain, where each node is an equal relationship. As a result, the back-end network can be jointly maintained by several different service providers and regulators, and these nodes check and balance each other, thus preventing a single service provider from privately collecting user data.

#### Protocols for the personal data protection mechanism

The personal data protection mechanism contains three protocols. The web application that satisfies the protocols can only be published and run on the PDP platform. Protocols are as follows:

**Protocol 1.** Prohibit the web front-end from communicating through existing network protocols, and the web front-end can only communicate with the web back-end deployed in the PDP back-end network through the API interface provided by the PDP front-end. This protocol can compulsorily constrain the web back-end to operate in the decentralized PDP back-end network, preventing the web front-end from privately transmitting user data to the service provider's servers.

**Protocol 2.** The web front-end contains a list of data tags describing all the user data that the web application will collect. The tag list is denoted as $\left\{tag_{i},description_{i}\right\}_i^w$, where *tag*_*i*_ is the tag name and $description_{i}$ is the description of the tag. The data collected by the web application must have one of the tags in the tag list, and the PDP front-end will generate the data stores and symmetric keys according to this tag list and manage the data and keys in each data store. Each data store is responsible for storing the data for each tag, and the symmetric key is responsible for encrypting and decrypting this data.

**Protocol 3.** The web front-end provides a data trading page, includes a smart contract on data trading, and inherits the built-in smart contract from the PDP back-end network, shown in Table 1. Through this protocol, the web application is responsible for setting the trading rules for user data. The user interacts with the smart contract on the web back-end through the data trading page on the web front-end according to the trading rules to realize trading their data on the blockchain.

**Table 1:** Functions of data trading contracts built into the PDP back-end network.

Data trading smart contract function	Function description
$\mathrm{tx}\_\mathrm{commision}(\alpha )$	Called by the service provider, the revenue sharing between the user and the service provider when setting trading data
$\mathrm{tx}\_\mathrm{request}(tx_{data})$	Called by the data purchaser to initiate a data trading request
$\mathrm{tx}\_\mathrm{request}(resp_{tx\_request},id_{tx\_request})$	Called by the user to respond to the trading request of the data purchaser
$\mathrm{tx}\_\mathrm{confirm}({id}_{tx\_response})$	Confirm the trading when the data buyer receives the user data

### Operation flow of the system

The personal data protection mechanism mainly contains three sub-mechanisms: data storage mechanism, data encryption mechanism, and data trading mechanism. The phases of the system operation can be divided into system initialization, data storage, data encrypted upload, and data trading. The data storage mechanism is responsible for storing the interaction data; the encryption mechanism is responsible for encrypting and uploading user data; and the data trading mechanism is responsible for issuing and confirming data trading requests. The following section describes each phase and mechanism of the system operation.

#### System initialization

**Figure 4:**
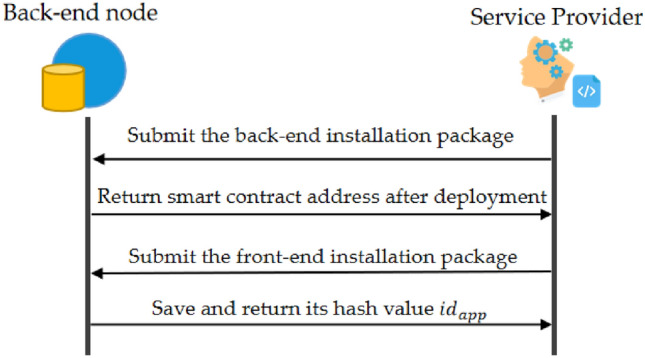
Application deployment.

The application deployment process is shown in Fig. 4. The service provider first submits the web back-end installation package to the PDP back-end network, parses the packet, deploys its program code to the blockchain as a smart contract, and then returns the smart contract address. The service provider sets the web front-end configuration file according to the smart contract address, realizes the web front-end and back-end communication, and then submits the web front-end program installation package to the PDP back-end network. The PDP back-end network saves the web front-end installation package in the distributed hash table. It returns the installation package's hash value as the web application's identity id.

**Figure 5:**
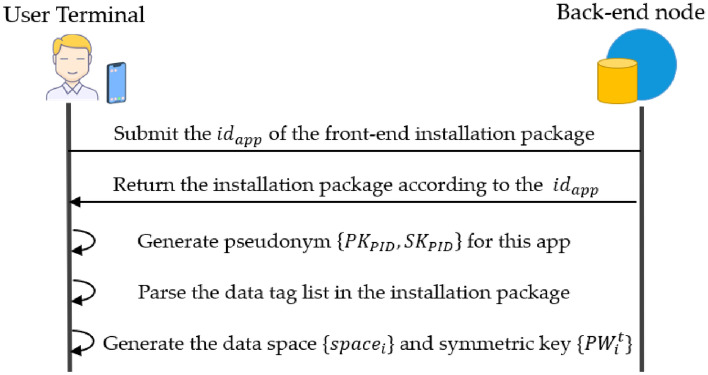
The PDP front-end initialization.

The PDP front-end initialization is shown in Fig. 5. When the user downloads the application, the PDP front-end will use the identity ${id}_{app}$ of the web application to download the installation package from the PDP back-end network and initialize it. To protect user privacy, the PDP front-end first generates a pseudonym PID for the user, whose public–private key pair is represented as $\left\{{PK}_{PID},{SK}_{PID}\right\}$. Then the data tag list in the web front-end is parsed to obtain the data tag set. The storage space and symmetric key are generated according to the tag set, denoted as ${\left\{{tag}_{i},{space}_{i},{PW}_{i}^{t,t+1}\right\}}_{t=1}^{w}$. Each ${tag}_{i}$ corresponds to a ${space}_{i}$ and corresponds to only one symmetric key ${PW}_{i}^{t,t+1}$ at any period $(t,t+1]$. Among them, ${PW}_{i}^{t,t+1}$ is time-sensitive and is responsible for encrypting the data located in the data storage space ${space}_{i}$ for a specific period $(t,t+1]$ during use to ensure the security of the data.

#### Data storage mechanism

**Figure 6:**
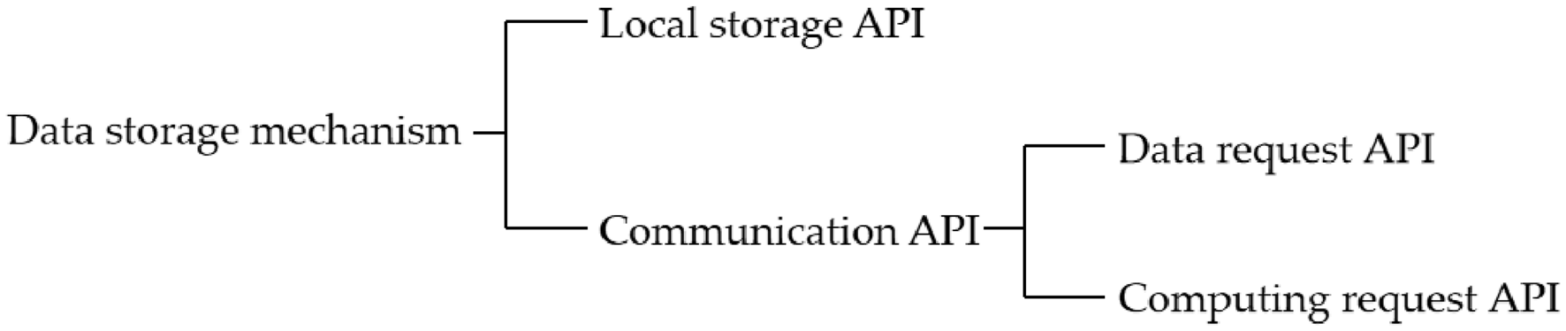
Data storage mechanism.

The data storage mechanism has two functions: (1) storing the data generated by the web front-end to the local storage space of the user terminal; (2) When the web front-end needs to communicate with the web back-end, it records the flow direction of the user data in the communication process. To realize the above functions, as shown in Fig. 6, the PDP front-end based on this mechanism provides two types of Application Programming Interfaces (APIs): local storage API and communication API.

**Figure 7:**
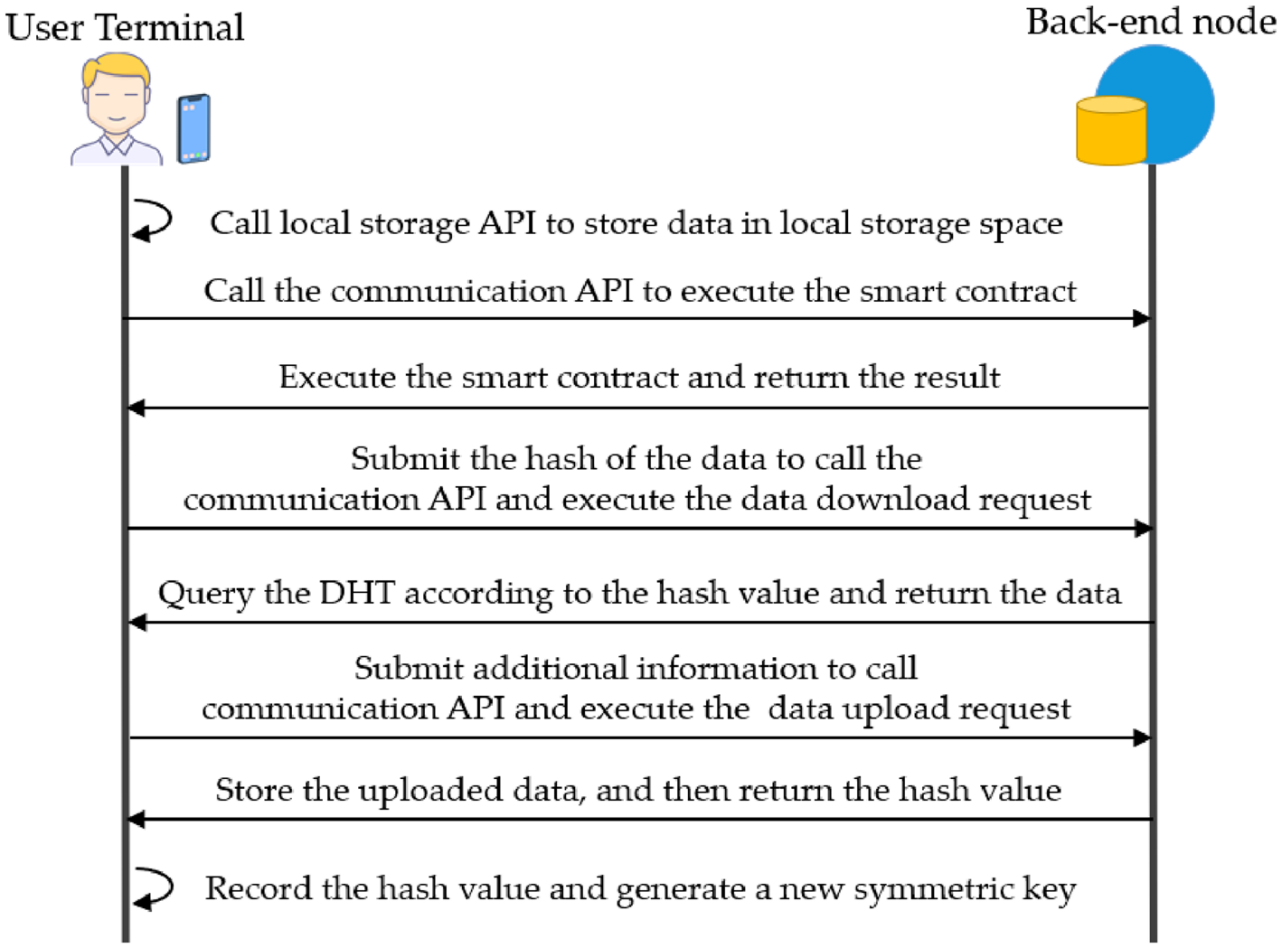
Data storage process.

These two types of APIs can meet the data storage and synchronization requirements during the operation of daily web applications. When the web front-end needs to collect user interaction data, it can use the local storage API to store the data in the local storage space of the user terminal. When the web front-end needs to communicate with the web back-end to synchronize data, it can use the communication API to communicate. The computation request API is responsible for requesting the PDP back-end to execute smart contracts. The data request API is responsible for uploading or downloading data to the PDP back-end network request. From the functions of these two types of APIs, it is clear that the communication API is the only channel for the web front-end to communicate with the external network. To prevent some malicious web front-ends from exposing users' original personal data (i.e., plaintext) to external networks through the communication API, whenever the web front-end invokes the communication API, the data storage mechanism will automatically record the invocation record in the blockchain, which is easy to trace and monitor the flow of user data. The overall flow of the data storage mechanism is shown in Fig. 7.

#### Encryption mechanism

In the data storage stage described above, the user data collected by the web front-end can only be stored in the local storage space, which will increase the local storage pressure of the user terminal. This paper designs the data encryption upload stage to relieve the local storage pressure. This phase is responsible for encrypting and uploading local user data to the PDP back-end network, thus relieving the local storage pressure of user terminals and facilitating users to trade their data subsequently. In the data encryption upload phase, user data will be uploaded to the PDP back-end network maintained by multiple untrusted nodes. It is essential to ensure the security of these uploaded data, for which an encryption mechanism is designed.

Symmetric encryption uses a single key to encrypt and decrypt a message, and the key must be distributed before the message is transmitted. Symmetric encryption algorithms are fast, less computationally intensive, and suitable for handling large amounts of data^27^. Common symmetric encryption algorithms include DES, 3DES, and AES. DES is a block cipher that uses a key length of 56 bits in actual use. If an attacker uses exhaustive methods to crack encrypted data, the number of operations is 2 to the 56th power. 3DES is an improved algorithm based on DES, which mainly encrypts a group of data in three rounds. Each round of encryption uses different keys, thereby enhancing the security of the data. AES encrypts plaintext by first grouping the plaintext, each group being 128 bits in length, and then encrypting it one group at a time until all of the plaintext has been encrypted. The length of the key can be 128, 192, or 256 bits. Compared to DES and 3DES, AES has higher security, faster computing speed, and less resource consumption. Overall, this article adopts the AES encryption algorithm for data encryption.

**Figure 8:**
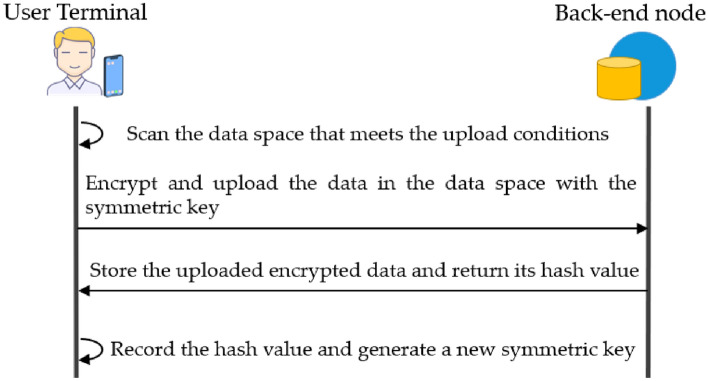
Data encryption process.

As shown in Fig. 8, in the encryption mechanism, the PDP front-end will scan the data in the data storage space at regular intervals. When the data storage space ${space}_{i}$ meets the upload condition, the symmetric key ${PW}_{i}^{t,t+1}$ is found according to the set ${\left\{{tag}_{i},{space}_{i},{PW}_{i}^{t,t+1}\right\}}_{t=1}^{w}$, and the data in ${space}_{i}$ is encrypted using this key. The DHT of the PDP back-end network stores the data and returns the hash value ${hash}_{i}^{t,t+1}$ to the encrypted data. The PDP front-end uses the hash value to form a new set ${\left\{{tag}_{i},{space}_{i},\left\{{PW}_{i}^{t,t+1},{hash}_{i}^{t,t+1}\right\}\right\}}_{i=1}^{w}$ and store it, while generating a new symmetric key ${PW}_{i}^{t+1,t+2}$ that will be responsible for encrypting the data for the next period $(t+1,t+2]$, and the data hash is generated as follows:

1 $$\begin{array}{c}{hash}_{i}^{t,t+1}=Hash\left({Encrypt}_{{PW}_{i}^{t,t+1}}\ \left({Data}_{{space}_{i}}\right)\right)\end{array}$$

#### Data trading mechanism

Under the role of data storage mechanism and encryption mechanism, the user data collected by the web front-end will be stored in the local storage space of the user terminal, eventually encrypted and uploaded to the PDP back-end network. These user data will be controlled by the user only, so the data purchaser can only buy its data from the user. For this situation, this paper designs a data trading mechanism.

**Figure 9:**
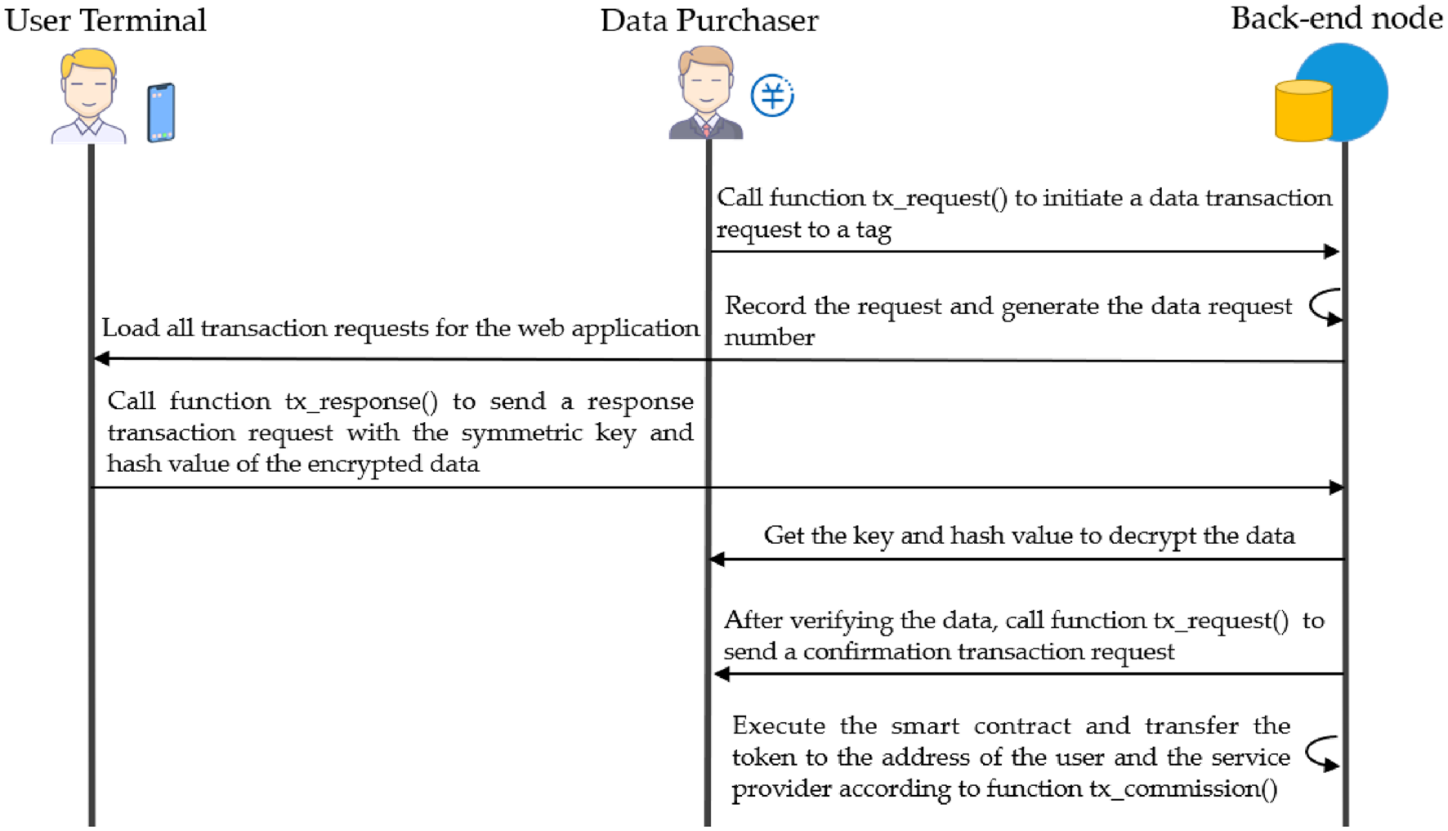
Data trading process.

As shown in Fig. 9, in the data trading mechanism, the data purchaser can obtain the tag list of each web application from the PDP back-end network, and select the web application to initiate a request to purchase data under a certain tag ${tag}_{i}$ by calling the function $\mathrm{tx}\_\mathrm{request}({tx}_{data})$ of the data trading smart contract of that web application with the following input parameters.

2 $$\begin{array}{c}{tx}_{data}=\left({tag}_{i}||{period}^{t,t+1}||{PK}_{C}||{price}_{i}\right)\end{array}$$

The request contains the tag ${tag}_{i}$ of the data to be purchased, the time period ${period}^{t,t+1}$ of the data generation, the public key ${PK}_{C}$ of the data purchaser and the purchase price ${price}_{i}$. The PDP back-end network publishes the trading request to the blockchain and generates the data request number ${id}_{tx\_request}$. The user using the web application can view this trading request information in the data trading page of the web front-end. When the user confirms the trading, the web front-end will call the smart contract function $\mathrm{tx}\_\mathrm{response}({resp}_{tx\_request},{id}_{request})$ to send a response message to the PDP back-end network with the following response.

3 $$\begin{array}{c}{resp}_{tx\_request}=({Encrypt}_{{PK}_{C}}({PW}_{i}^{t,t+1})||{hash}_{i}^{t,t+1})\end{array}$$

The user response will generate the response number ${id}_{tx\_request}$, and the data purchaser can use its private key ${SK}_{C}$ to decrypt the data to obtain the corresponding symmetric key ${PW}_{i}^{t,t+1}$, and then download the corresponding data from the back-end network according to ${hash}_{i}^{t,t+1}$, and finally use ${PW}_{i}^{t,t+1}$ to decrypt the data and obtain the user data. After the data requesting organization confirms the trading, it calls the smart contract function $\mathrm{tx}\_\mathrm{confirm}({id}_{response})$ to the PDP back-end network to complete the data trading. The data requesting organization pays the corresponding token ${token}_{i}$ for the data, and the smart contract will transfer the tokens $\alpha *{token}_{i}$ and $\left(1-\alpha \right)*{token}_{i}$ to the user and service provider, respectively, according to the revenue sharing ratio set in the function $\mathrm{tx}\_\mathrm{commision}(\alpha )$ both the user and the service provider can get revenue from the data trading.

## Results and discussion

### AnyValueEmail's operation process

To illustrate how the personal data protection mechanism can ensure the security of users' data, use a web application as an example to show its process on PDP. It analyzes how the mechanism can protect users' data during web application. This web application, called AnyValueEmail, is an email application that differs from ordinary email. It ensures that the service provider cannot collect users' data arbitrarily.

AnyValueEmail is developed by third-party developers to be deployed to run on PDP according to the specification. The front-end of AnyValueEmail needs to contain a list of data tags that include all types of user data, such as the content of sent emails, the content of received emails, email attachments, etc. The following is an example of the content record tag ${tag}_{publish}$ when sending emails to analyze the specific operation process. In addition, AnyValueEmail includes a data trading smart contract in its back-end. The service provider sets the data revenue sharing ratio by calling the function $\mathrm{tx}\_\mathrm{commision}(\alpha )$ in this contract, and for simplicity, the revenue sharing ratio $\alpha$ is set to 0.5.

**Figure 10:**
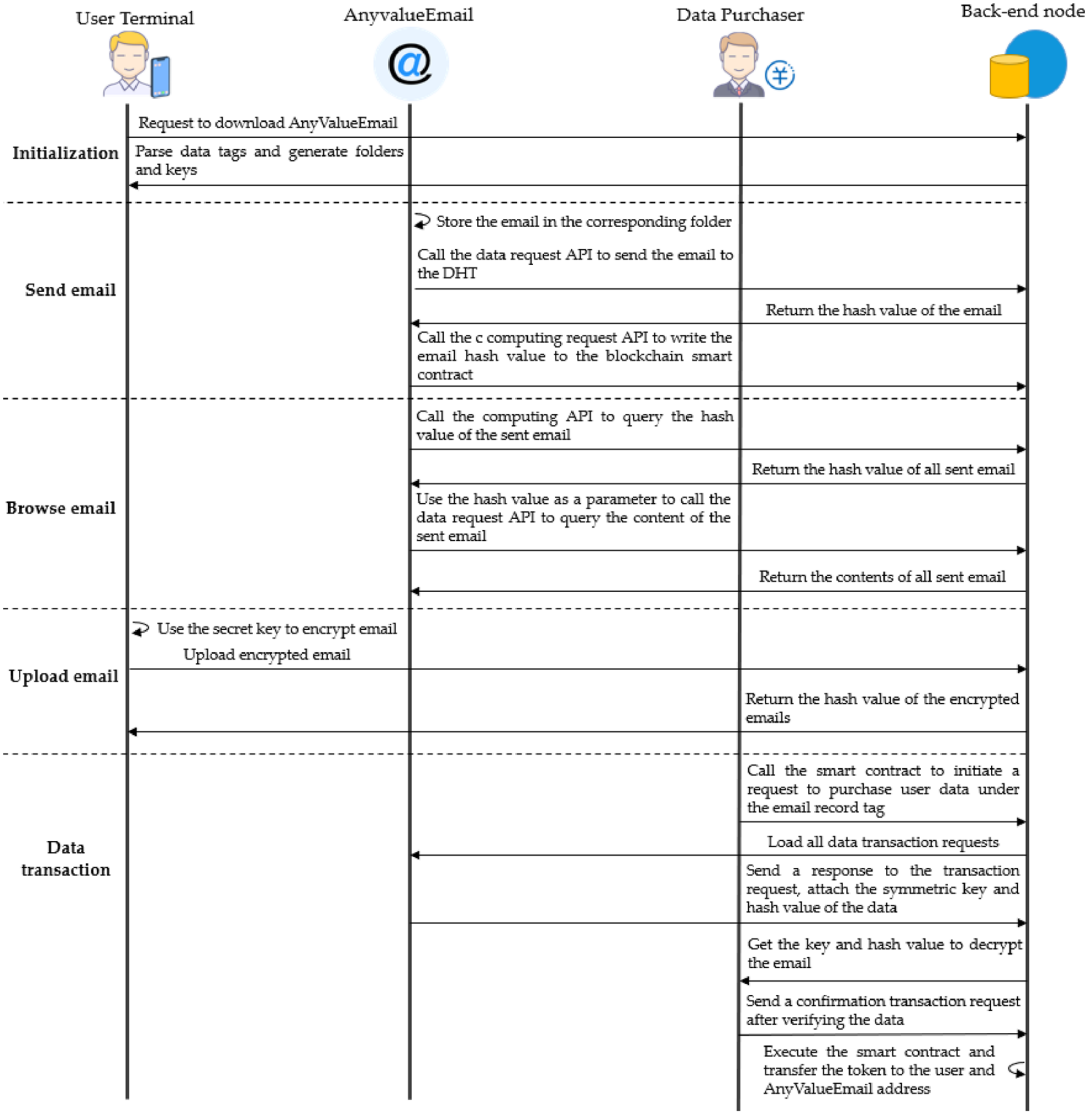
AnyValueEmail's operation process.

Using the AnyValueEmail application, since the sender's identity and the content of the email are encrypted, no one else can access the email and any information about the two users except the two parties to the email. The information exchanged between the two sides of the email (i.e., the email record corresponding to the data tag ${tag}_{publish}$) is stored as personal data in the local storage of user, who can get hold of that data. Suppose other third parties need to be informed about the information related to the emails in AnyValueEmail. In that case, they need to purchase the user data under the tag ${tag}_{publish}$ from the owner of the email. Specifically, the flow of a user posting an email is shown in Fig. 10.

### Security analysis of AnyValueEmail

There may be a risk of AnyValueEmail using data request API and computing request API to leak users' data to external networks. However, in real-world applications, sensible AnyValueEmail service providers choose to assist users in protecting their privacy. Because when AnyValueEmail uses the above APIs, its actions are recorded and traceable, and all data uploaded to the external network is public to all nodes, according to the data storage mechanism. When AnyValueEmail does not protect users' data and leaks their private data, on the one hand, all nodes have access to this leaked information, and AnyValueEmail service providers can no longer obtain additional revenue by monopolizing users' data. On the other hand, when regulators and users monitor AnyValueEmail in the first place, AnyValueEmail may face the risk of being taken down by regulators and abandoned by users when regulators and users first monitor that AnyValueEmail has compromised user privacy.

In the above process, the AnyValueEmail application does not benefit from disclosing user data but causes losses. In contrast, AnyValueEmail can benefit more from protecting user privacy. For example, suppose AnyValueEmail anonymizes the user's identity at the time of posting. In that case, the external network cannot learn the user's record of sending emails. When the data purchaser needs to use the data for research, it can only buy data from the user through a smart contract under the tag ${tag}_{publish}$, then gets a share of the revenue from that trading process. Based on the PDP mechanism, the AnyValueEmail application has a positive benefit-driven approach to providing services to users on PDP to protect user data security and prevent personal privacy leakage practically.

### Data trading revenue optimization

#### Stackelberg game

As a commonly used game model, the Stackelberg Game reacts to asymmetric games in competitive markets. It is an imperfect competition model based on a noncooperative game^28^, which assumes the existence of dominance and followers in the market. The market positions of the dominant and the follower are asymmetric, affecting both parties' decision sequences. Usually, the dominant player makes a decision first, and the follower observes the dominant player's decision before deciding. Under the Stackelberg model, the dominant anticipates that the followers will adjust their decisions according to the dominant's decisions, so they need to consider the followers' reactions when making decisions.

#### Data trading revenue optimization based on the Stackelberg game

The revenue from data trading is optimized by optimizing this revenue sharing ratio $\alpha$ to maximize the revenue of users and service providers. This paper uses the Stackelberg game to solve the above problem. In this game, the service provider and the user adjust each other according to the revenue sharing ratio and the trading data volume until the Stackelberg equilibrium is finally achieved and the revenue of both parties is maximized.

After the service provider publishes the web application to PDP platform, the user downloads the web application to the user terminal and generates the user data. of using it. At the same time, the amount of data sold ${x}_{i}$ is determined according to the revenue sharing ratio $\alpha$ set by the service provider. Where $\alpha$(0 ≤ $\alpha$  ≤ 1) refers to the revenue percentage of the user after selling the data, and for every revenue gained by selling the data, the user gets the revenue percentage $\alpha$ and the service provider gets the remaining percentage (1 − $\alpha$).

1. Revenue function analysis.

The set of users using the web application provided by the service provider is denoted as $I$. The revenue function of a user in the set $I$ when selling data according to the revenue sharing ratio is as follows:

4 $$\begin{array}{c}{U}_{i}={\lambda }_{i}ln\left(\alpha \tau {x}_{i}+1\right)-{\mu }_{i}{x}_{i}\end{array}$$

where ${x}_{i}$ is the user's decision variable, representing the amount of data that user will sell, and since data can be sold repeatedly, there is a case where ${x}_{i}>{X}_{i}$; ${\lambda }_{i}$(${\lambda }_{i}>0$) is a predefined parameter, representing the user's willingness degree; ${\mu }_{i}$ represents the unit overhead of the user when selling data^29^; $\tau$ represents the price per unit of data. As the slave of the game, the user will adjust the amount of data sold to maximize its revenue according to the percentage $\alpha$ set by the service provider.

The service provider's data revenue mainly comes from the revenue share in user-traded data. In this mechanism, since the service provider does not need to maintain the user-generated data, its cost is the initial investment cost. The specific revenue function is as follows:

5 $$\begin{array}{c}{U}_{sp}=\left(1-\alpha \right)\tau \sum_{i\in I}{x}_{i}-C\end{array}$$

As the game master, the service providers offering data collection services aim to maximize the revenue from data trading by setting an appropriate revenue share.

(2) Stackelberg equilibrium solution.

In this paper, the backward derivation method is used for the Stackelberg equilibrium solution, which first analyzes the optimal return of the user under the return ratio $\alpha$. The first and second order partial derivation of Eq. (4), we get:

6 $$\begin{array}{c}\frac{\partial {U}_{i}}{\partial {x}_{i}}=\frac{\alpha \tau {\lambda }_{i}}{\alpha \tau {x}_{i}+1}-{\mu }_{i},\end{array}$$

7 $$\begin{array}{c}\frac{{\partial }^{2}{U}_{i}}{\partial {x}_{i}^{2}}=-\frac{{\left(\alpha \tau \right)}^{2}{\lambda }_{i}}{{\left(\alpha \tau {x}_{i}+1\right)}^{2}}<0.\end{array}$$

According to formula (7), ${U}_{i}$ is a strictly concave function, which ensures the existence of unique solutions. Therefore, we can make $\frac{\partial {U}_{i}}{\partial {x}_{i}}=0$, so that the proportion of data sold when the user's best income is obtained is:

8 $$\begin{array}{c}{x}_{i}^{*}=\frac{{\lambda }_{i}}{{\mu }_{i}}-\frac{1}{\alpha \tau }.\end{array}$$

Substitute formula (8) into the revenue function formula (5) of the main side of the game, and the result is:

9 $$\begin{array}{c}{U}_{sp}=\left(1-\alpha \right)\tau \sum_{i\in I}\frac{{\lambda }_{i}}{{\mu }_{i}}-\frac{1}{\alpha \tau }-C.\end{array}$$

Performing the first and second order partial derivatives of Eqs. (9), we get:

10 $$\begin{array}{c}\frac{{\partial U}_{sp}}{\partial \alpha }=\sum_{i\in I}\left(-\frac{{\tau \lambda }_{i}}{{\mu }_{i}}+\frac{1}{{\alpha }^{2}}\right),\end{array}$$

11 $$\begin{array}{c}\frac{{\partial }^{2}{U}_{sp}}{\partial {\alpha }^{2}}=-\frac{2\left|I\right|}{{\alpha }^{3}}<0,\end{array}$$

where $|I|$ refers to the number of users in the set $I$. According to Eq. (11), it is known that the ${U}_{sp}$ is a concave function that ensures the existence of a unique solution. In this mechanism, when ${x}_{i}^{*}=0$ for user $i$, it often means that the user is more sensitive to personal privacy and does not want to sell his personal data. Therefore, the service provider can consider that this user *i* is not its target user, so it is not necessary to consider all users when calculating the revenue function of the game master. From Eq. (8), we know that ${x}_{i}^{*}>0$ when $\alpha >\frac{{\mu }_{i}}{\tau {\alpha }_{i}}$, so this paper only considers the case when $\alpha >\frac{{\mu }_{i}}{\tau {\alpha }_{i}}$.

(3) Stackelberg equilibrium using an iterative optimization algorithm.

**Figure 11:**
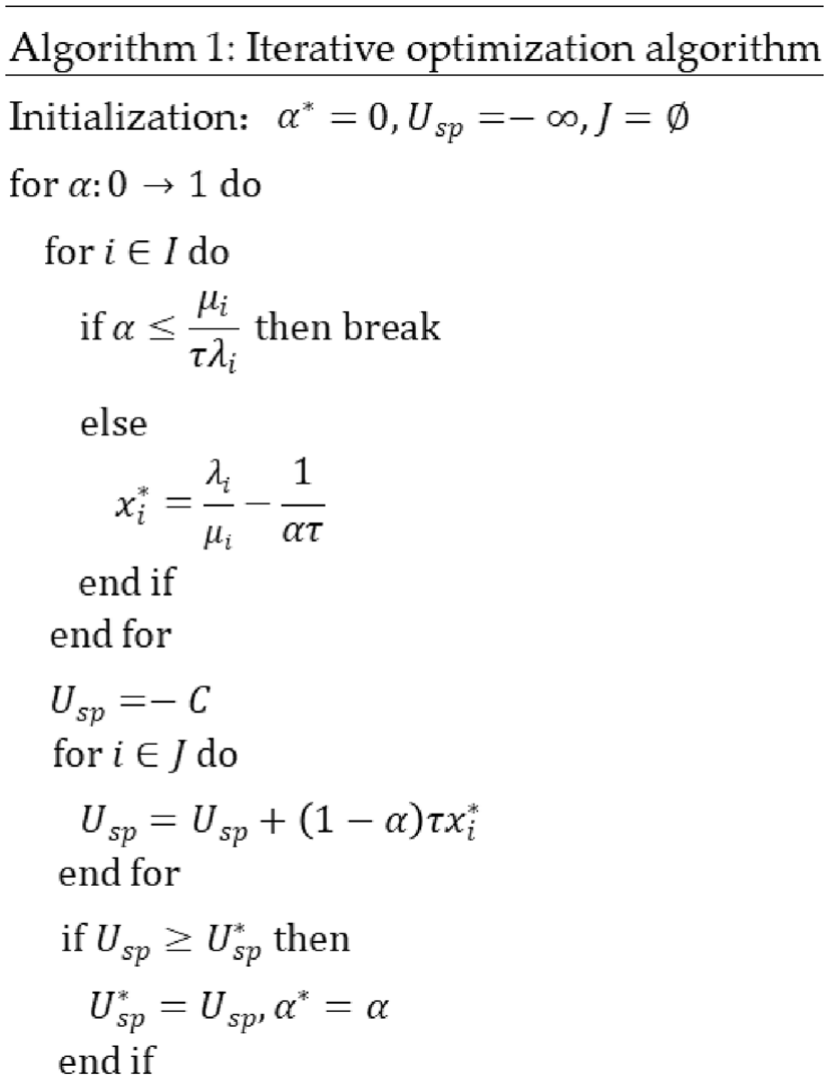
Data trading iterative optimization algorithm.

We propose to use an iterative optimization-seeking method to achieve the solution of Stackelberg equilibrium, and the process is shown in Fig. 11. Through multiple rounds $\alpha$ dynamically changes to gradually increase the step size of $\Delta \alpha$ from 0 to 1. In each round, the service provider on the main side passes the dynamic $\alpha$ value to each user, who gives the best response at this time according to Eq. (8); subsequently, the service provider collects all responses satisfying $\alpha >\frac{{\mu }_{i}}{\tau {\lambda }_{i}}$ to calculate the current own revenue value to determine whether it is better than the previous recorded optimal revenue value, and if so, replaces the previous optimal revenue value with the current revenue value If yes, replace the previous optimal revenue value with the current revenue value and update ${\alpha }^{*}$. In this way, the service provider can obtain the best response ${\alpha }^{*}$ without disclosing any personal information to the user, and then deterministically release it to the user, so that the user also outputs a stable best response ${x}_{i}^{*}$. At this point, both parties maximize their revenue at the same time, and there is no incentive to change other strategies, and finally the game model reaches the Stackelberg equilibrium.

#### Simulation and analysis

Referring to the literature^30^, the values of the parameters set in the simulation experiments of this paper are shown in Table 2, where the parameters in the range of values obey a uniform distribution. From the perspective of maximizing the service provider's revenue, the following three options are available: (1) Equal sharing of revenue. From the fairness perspective between users and service providers, the revenue of user data trading is equally distributed, $\alpha =$ 0.5; (2) The proportion of revenue positively correlates with data volume. Out of the perspective that the larger the data market size is, the more the service provider gives away, the proportion of revenue share that users get from data trading increases in positive proportion to the overall user data volume, $\alpha =a{\sum }_{i\in I}{X}_{i}+b$; (3) The proportion of revenue is negatively correlated with data. Out of the perspective that service providers encourage users to trade data and cultivate the data trading market in the early stage, the proportion of revenue share obtained by users from data trading decreases positively to the overall user data volume, $\alpha =c-a{\sum }_{i\in I}{X}_{i}$.

**Table 2:** List of simulation parameters and values.

Simulation parameters	Numerical range
$a$	8 × 10^–6^/M bytes
$${\lambda }_{i}$$	[10, 100]
$${\mu }_{i}$$	[0.1, 2]
$\Delta \alpha$	0.02
$\tau$	0.1/M bytes
$C$	100
Number of users	[100, 1000]
Initial value of $\alpha$ in Option 2	0.1
Initial value of $\alpha$ in Option 3	0.9
Range of $\alpha$ in the iterative algorithm	[0, 1]

**Figure 12:**
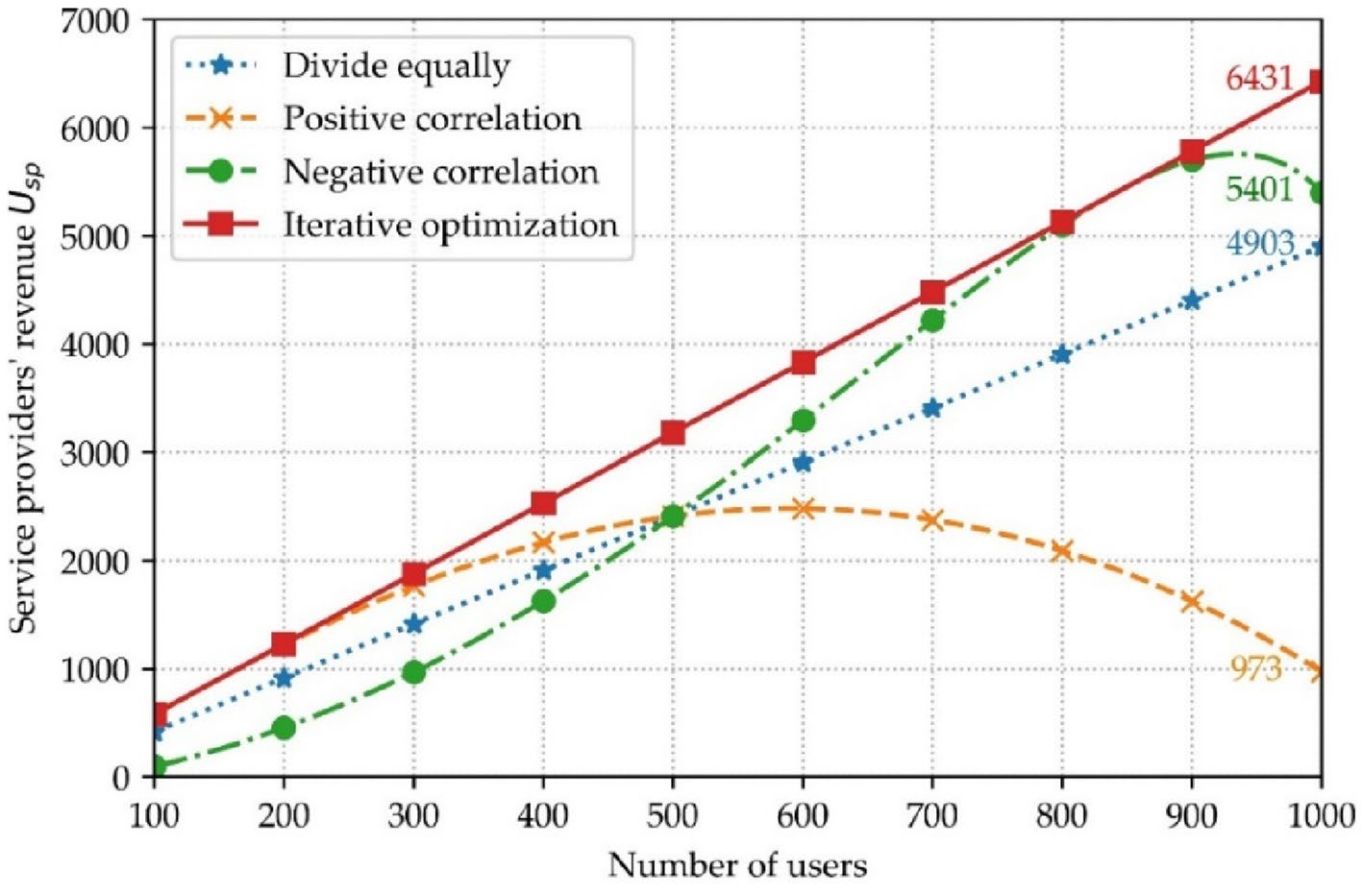
Comparison of schemes.

Figure 12 shows the comparison between the iterative optimization algorithm and the above three schemes regarding the revenues obtained by the service provider in user data trading. The revenues received by the service provider in the data trading process of the iterative optimization algorithm are better than the other schemes for the different numbers of users. When the number of users is 1000, the revenues of the iterative optimization algorithm for the service provider are 31%, 561%, and 19% higher than the existing schemes, respectively.

**Figure 13:**
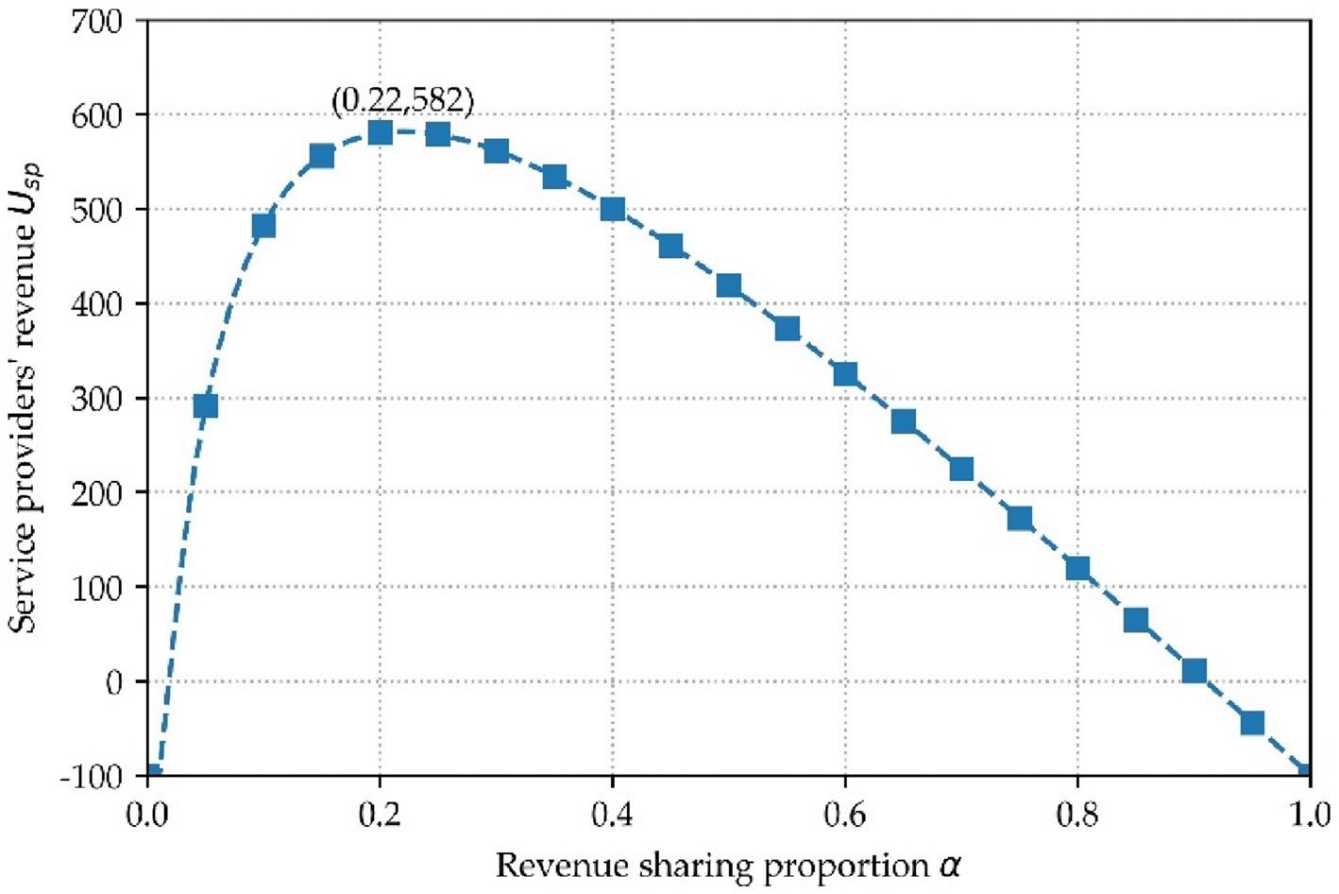
Relationship between α and ${U}_{sp}$.

Figure 13 shows the service provider's revenue for different revenue sharing ratios when the number of users is 100. It can be seen from the figures that different revenue sharing ratios lead to different amounts of data that users are willing to sell, which ultimately affects the revenue of the service provider in data trading. It also demonstrates that the iterative search algorithm in this paper can find the optimal revenue sharing ratio α through iterative computation.

**Figure 14:**
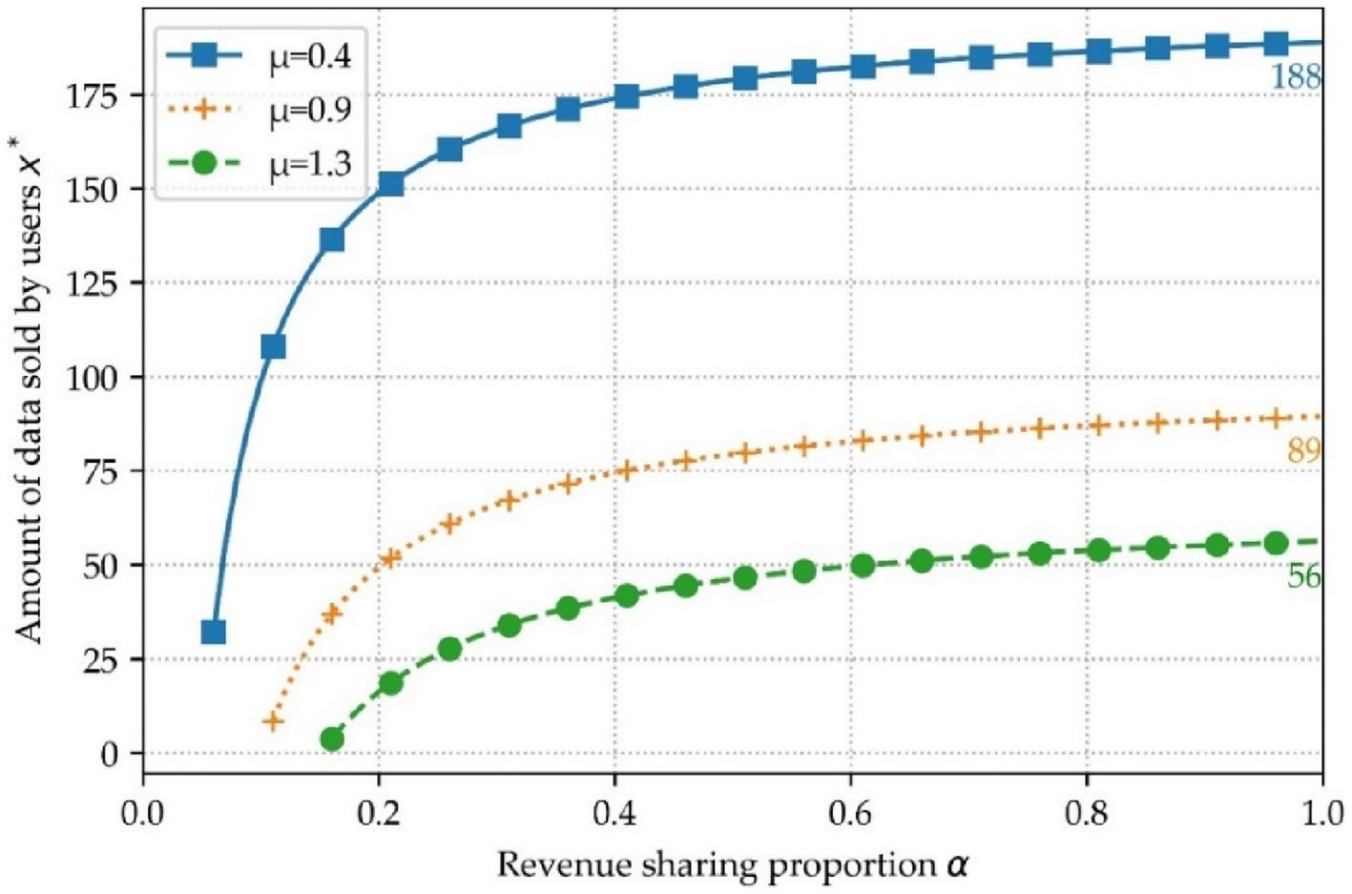
Relationship between α and μ.

In Fig. 14, the optimal amount of data for sale is randomly selected for one user. The figure shows the negative effect of the unit trading data overhead μ on the user's optimal amount of data for sale, i.e., when μ is larger, the less the user wants to sell his or her personal data. Also, Fig. 14 illustrate that the revenue sharing ratio α can serve as an incentive for users to sell their personal data.

It can be shown in the simulation experiments that the Stackelberg game-based optimization scheme used in this paper is significantly better than other schemes in the process of data trading. The data revenue of users and service providers is maximized.

## Design of the platform

### Design of file structures and data structures

Since the PDP platform is a runtime environment for running multiple third-party web applications, it needs to develop some protocols. web applications that satisfy the protocols can run on the platform, so before designing the overall framework of the PDP plotform, this chapter will discuss the platform's requirements for the file structure and data structure of web applications.

#### Frontend installation package file directory

**Figure 15:**
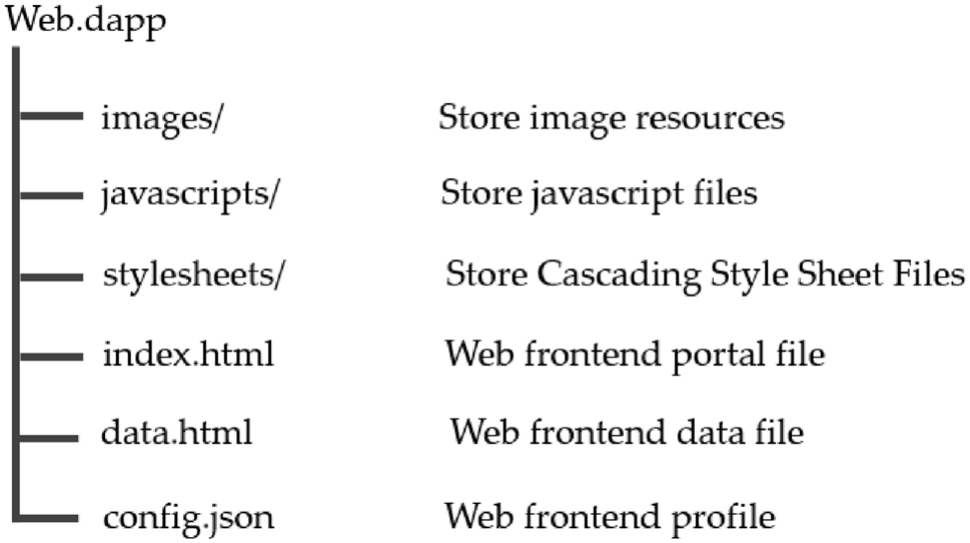
Web front-end installation package file structure.

The file structure of the web front-end installation package is shown in Fig. 15, which shows the role of each subdirectory, among which the main files are config.json, index.html, and data.html. The config.json file records the configuration information of the web front-end, including the list of its data tags. The PDP front-end will first parse this configuration file and initialize the web front-end according to this configuration file. The index.html file is the entry file of the web front-end, which will be loaded first when the PDP front-end runs the web front-end. data.html file is the data file of the web front-end, which will be loaded when the user enters the data interface of the PDP front-end for data trading. The data.html file is the data file of the web front-end.

#### Tag list data structure

The tag list data structure is as described above. The config.json file stores the tag list information of the web front-end, and the tag list records the tags of the data that the web front-end will collect. When initialization, the PDP front-end will parse the tag list and generate the data storage space and symmetric key for each tag. When the web front-end calls the API to store the data, it must also provide the tags to store it in the storage space.

#### Data structure of the key system

After the PDP front-end parses the web front-end installation package, it maintains a data structure to manage all the tags of the web front-end and their corresponding key systems, which records all the encrypted data information of the web front-end.

The data structure consists of three parts: the identity ${id}_{app}$ of the web application, the user pseudonym PID and the symmetric key PW corresponding to the data tag. In this paper, considering the user's need to change the pseudonym at any time, we design an array of pseudonyms and set the enable time and expiration time for each pseudonym, where when the expiration time is empty, it means that the pseudonym is in use. And because symmetric keys are responsible for encrypting personal data generated by users over a period of time, this paper also designs a symmetric key array, and each time period corresponds to a symmetric key set. The symmetric keys under each tag are responsible for encrypting the data in its storage space. The index information of the uploaded encrypted data is recorded, and easy to retrieve data with the PDP back-end network.

### Operation process and interface of the platform

The PDP platform mainly consists of the PDP front and back-ends. The platform needs to provide the necessary basic functions for users to use the web applications. It also needs to provide the API for the web applications running on the platform so that the web applications can interact with the platform. This section briefly introduces the data encryption process and the interaction API.

**Figure 16:**
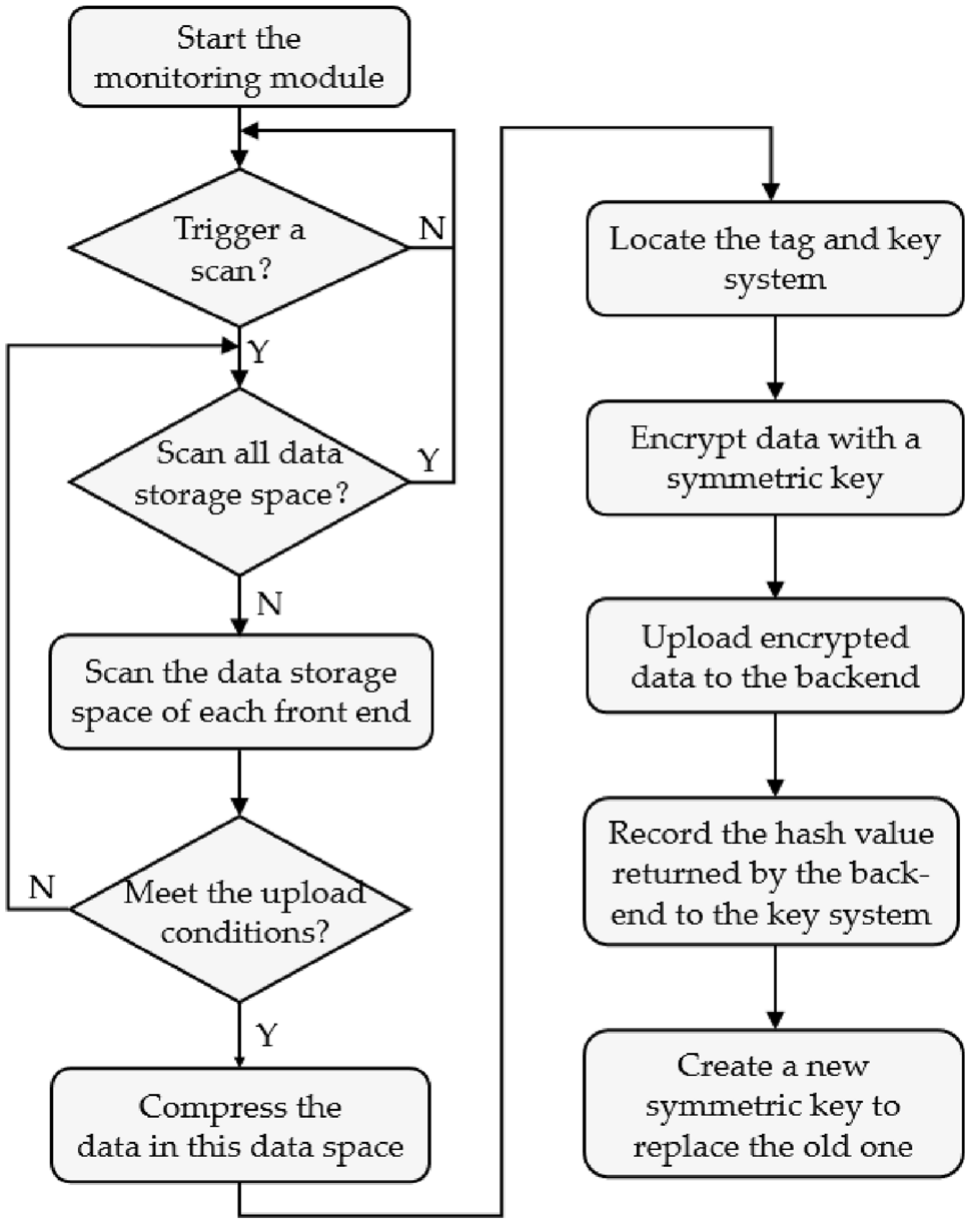
Flowchart of data encryption upload.

The data encryption and uploading process are shown in Fig. 16. In user use, the PDP front-end will scan the data storage space of all installed web front-ends at regular intervals. When the data storage space meets the upload conditions, the PDP front-end will compress, encrypt and upload the data in that space and update the symmetric key and index information in the key system. The data encryption and uploading process are shown in Fig. 16. In user use, the PDP front-end will scan the data storage space of all installed web front-ends at regular intervals. When the data storage space meets the upload conditions, the PDP front-end will compress, encrypt and upload the data in that space and update the symmetric key and index information in the key system.

The interaction APIs provided by the PDP front-end need to meet the requirements for properly operating the web application on the platform. The functions provided by the interaction APIs are shown in Table 3. By using these APIs, the web front-end can easily store the generated data in the local storage of the PDP front-end. It can also communicate with the web back-end deployed in the PDP back-end network.

**Table 3:** The Function of interaction API.

Function	Description
Remote computing request	Initiate a request to execute a smart contract in the PDP back-end network and obtain the execution result
Remote storage requests	Initiate requests to perform upload/download of data in the PDP back-end network
Local storage request	Query/store data to the local storage space on the PDP front-end based on data tags
Data trading requests	Initiate a request to execute a data trading contract to the PDP back-end network and obtain the execution result
Utility requests	Provide to meet the operational needs of the web front-end, such as installing web front-end requests, obtaining user pseudonym public key requests, etc

### The framework of the platform

**Figure 17:**
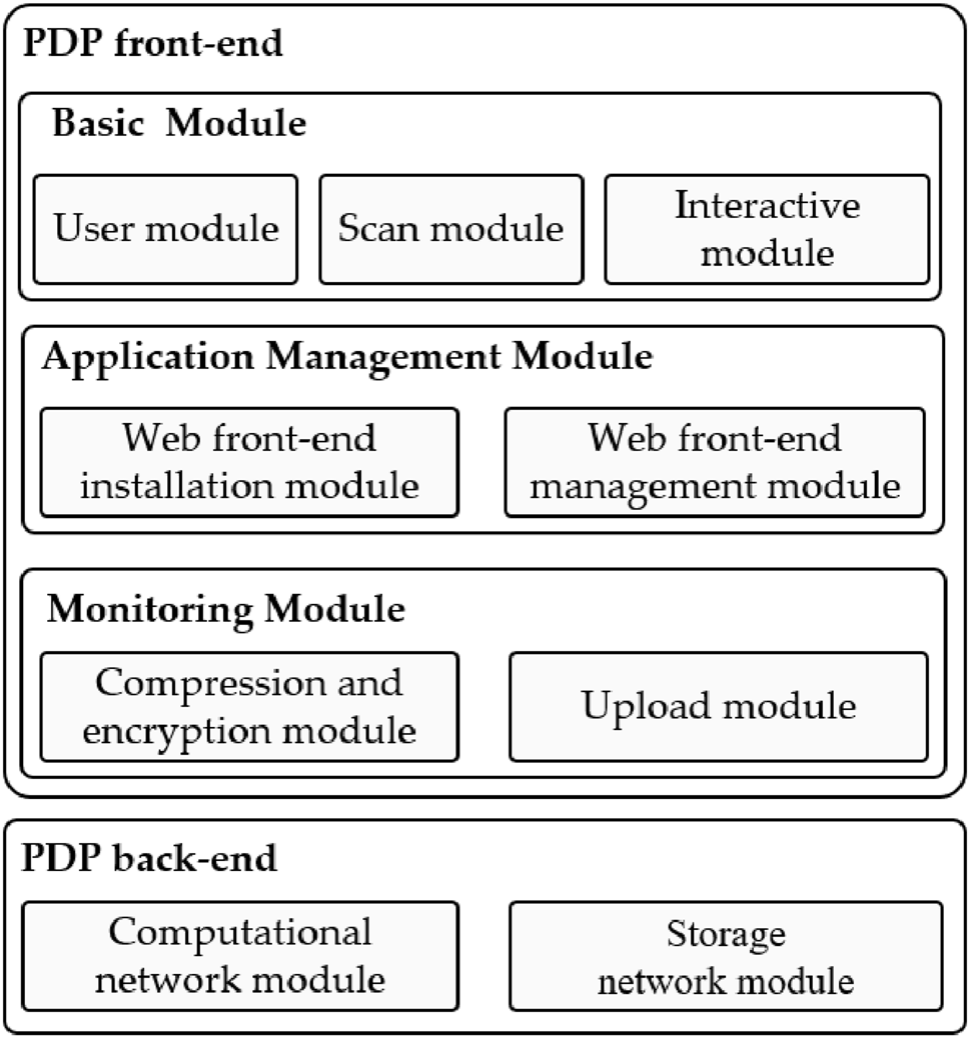
The framework of PDP platform.

The platform's framework is divided into two parts, the front-end and back-end network, as shown in Fig. 17, and the functions and connections of each module are divided in a modular way.

In the PDP front-end, the functions of each module are as follows: the user module is responsible for managing the user's local account information and key system; the QR code scanning module is responsible for scanning the QR code and identifying the hash value of the web front-end installation package from the QR code; the interaction module is responsible for providing the interaction API for the web front-end running in the PDP front-end, so as to ensure the normal operation of the web application; the application management module is responsible for managing all web front-ends, where the web front-end installation module is responsible for downloading the installation package of the web front-end from the PDP back-end network, and parsing and installing the application based on the information of the installation package, and the installed web front-end will be run and deleted by the web front-end management module; the monitoring module is responsible for monitoring the user data in the local storage space of the user terminal, and it will regularly monitor the data that needs to be encrypted and uploaded using compression and encryption. The monitoring module is responsible for monitoring the user data in the local storage space of the user terminal, which will regularly monitor the data to be encrypted and uploaded and then use the compression and encryption module to compress and encrypt the data.

The PDP back-end network is divided into the compute and storage network modules. The compute network module is built based on Ethereum. It integrates monitoring smart contracts and data trading smart contracts in this network, mainly providing compute requests for the PDP front-end. The storage network module is built based on IPFS and maintains a DHT in multiple peer-to-peer back-end network nodes to provide data requests for the PDP front-end.

## Conclusion and outlook

Based on blockchain technology, this paper designs a personal data protection mechanism. Prevent enterprises providing web services from privately collecting users' data, and ensure the personal data generated by users in using web applications are in the hands of users. The conclusions of the whole paper are as follows:

1. We design personal data protection mechanism. The system model faced by this research topic is induced from the practical problem to be solved, then design the protocols and sub-mechanisms of the mechanism in conjunction with the operation process of the system, and discuss how these protocols and sub-mechanisms act in the whole system.
2. Conducts a security analysis of the personal data protection mechanism and optimizes the revenue of data trading. In the security analysis, the privacy and security features protected by the mechanism are summarized, followed by an application example to analyze further how the mechanism protects users' data. In the data trading revenue optimization, the chapter uses the Stackelberg game to optimize the revenue of the data trading process and conducts simulations and analyses.
3. Analyzes the file structure, data structure, operation process, and interface implementation details of the platform, and then design the platform's architecture in the way of module division, clearly dividing the functions and relationships of each module.

During the research process, due to time, resources, and academic limitations, there are still the following issues:

1. The implementation of this research topic adopts Ethereum as the backend network computing platform. However, currently, Ethereum's computing power is limited, making it difficult to meet the practical needs of hundreds of thousands of accesses per second, which also restricts the practical application of the platform.
2. The back-end network of PDP is only briefly considered in this research question, which can be maintained by a number of different service providers and regulators, but does not discuss in depth the additional management overheads and communication overheads associated with a multi-node network.

In future work, the first step is to investigate how to improve the processing performance of the entire back-end network to cope with the demands of real-world usage. On this basis, a reasonable number of nodes in the back-end network is investigated to optimize the overall overhead of this network while ensuring data security.

## Data Availability

The datasets used and analyzed during the current study are available from the corresponding author upon reasonable request.
